# 540 Evaluation of Factors Contributing to Autologous Skin Graft Failure in the Outpatient Setting

**DOI:** 10.1093/jbcr/iraf019.169

**Published:** 2025-04-01

**Authors:** Kathryn Curtin, Elizabeth Shannon, Elisabeth Alison, Alexandra Alba, Bryn Kelly, Bonnie C Carney, Shawn Tejiram, Taryn Travis, Jeffrey Shupp, Lauren Moffatt

**Affiliations:** The Burn Center at MedStar Washington Hospital Center; Medstar Washington Hospital Center; Medstar Washington Hospital Center; Medstar Washington Hospital Center; The Burn Center at MedStar Washington Hospital Center; Medstar Washington Hospital Center; The Burn Center at MedStar Washington Hospital Center; The Burn Center at MedStar Washington Hospital Center; The Burn Center at MedStar Washington Hospital Center; MedStar Health Research Institute

## Abstract

**Introduction:**

Autologous skin grafts serve as the definitive coverage for lost or damaged skin and can be used on a variety of different wounds, including burns and complex dermatologic injuries unable to be repaired by primary closure. The universal objective in performing autologous skin grafting is for complete graft adherence without areas of significant graft failure, especially post-discharge. The purpose of this study is to identify what factors may contribute to post-discharge graft loss. It was hypothesized that there is more occurrence of graft loss in grafted burns with joint involvement, and also more graft loss in those patients who get discharged to a facility.

**Methods:**

The study was a retrospective chart review of patients who were admitted to a regional burn center and underwent autologous split thickness skin grafting (STSG) from January 2023 through August 2024 who had documented graft loss after their hospital discharge. Demographics, co-morbidities, grafting size and location, discharge disposition, type of dressing, and management strategies were evaluated.

**Results:**

Overall, 148 patients with post-discharge graft loss were included in this study. The average age was 47.61 years old (SD +/- 17.34). The average length of stay after the last graft application was 7.64 days (SD +/- 6.43). A majority (64.86%) of these patients received allograft or synthetic skin substitute prior to autologous skin. Over half (55.41%) of the patients had no joint involvement in the areas with graft loss. There was 47.30% of the population were discharged home independently without need of services. A majority of patients (60.14%) were discharged home in dressings that remained in place until follow up in clinic.

**Conclusions:**

Contrary to our hypotheses, a majority of patients with graft loss did not have joint involvement, and most patients included in the analysis were discharged home independently with dressings to remain in place and not be changed until the clinic visit. Further research will compare patients with and without graft loss to better clarify contributing factors that may be present in one group versus the other.

**Applicability of Research to Practice:**

If factors contributing to outpatient graft-loss can be identified, an intervention may be applied which can ameliorate graft loss in future patients.

**Funding for the Study:**

N/A

